# High Precision Bone Cutting by Er: YAG Lasers Might Minimize the Invasiveness of Navigated Brain Biopsies

**DOI:** 10.3389/fonc.2021.690374

**Published:** 2022-01-03

**Authors:** Thanh Tu Ha, Florian M. Thieringer, Martin Bammerlin, Dominik Cordier

**Affiliations:** ^1^ Department of Neurosurgery, University Hospital Basel, Basel, Switzerland; ^2^ Advanced Osteotomy Tools AG, Basel, Switzerland; ^3^ Department of Oral and Cranio-Maxillofacial Surgery, University Hospital Basel, Basel, Switzerland; ^4^ Medical Additive Manufacturing Research Group, Department of Biomedical Engineering, University of Basel, Allschwil, Switzerland

**Keywords:** diagnostic, brain lesion, Er : YAG laser, laser osteotomy, cold ablation, brain biopsies

## Abstract

Biopsies of brain tissue are sampled and examined to establish a diagnosis and to plan further treatment, e.g. for brain tumors. The neurosurgical procedure of sampling brain tissue for histologic examination is still a relatively invasive procedure that carries several disadvantages. The “proof of concept”-objective of this study is to answer the question if laser technology might be a potential tool to make brain biopsies less invasive, faster and safer. Laser technology might carry the opportunity to miniaturize the necessary burr hole and also to angulate the burr hole much more tangential in relation to the bone surface in order to take biopsies from brain regions that are usually only difficult and hazardous to access. We examined if it is possible to miniaturize the hole in the skull bone to such a high extent that potentially the laser-created canal itself may guide the biopsy needle with sufficient accuracy. The 2-dimensional, i.e. radial tolerance of the tip of biopsy needles inserted in these canals was measured under defined lateral loads which mimic mechanical forces applied by a surgeon. The canals through the skull bones were planned in angles of 90° (perpendicular) and 45° relative to the bone surface. We created a total of 33 holes with an Er : YAG laser in human skull bones. We could demonstrate that the achievable radial tolerance concerning the guidance of a biopsy needle by a laser created bone canal is within the range of the actual accuracy of a usual navigated device if the canal is at least 4 mm in length. Lateral mechanical loads applied to the biopsy needle had only minor impact on the measurable radial tolerance. Furthermore, in contrast to mechanical drilling systems, laser technology enables the creation of bone canals in pointed angles to the skull bone surface. The latter opens the perspective to sample biopsies in brain areas that are usually not or only hazardous to access.

## Introduction

In case of unclear brain lesions in imaging studies, tissue biopsies are mandatory for a definitive histologic diagnosis and adequate treatment planning, e.g. for brain tumors. In many of these cases, navigated brain biopsies are routinely performed by directing a biopsy needle in the brain’s affected area. Depending on the institution and the surgeon’s preferences, this kind of biopsy often includes a skin incision of up to more than 4 centimeters and a burr hole of up to 14 millimeters in diameter. Furthermore, an incision of the dura mater and the insertion of the biopsy needle, often through functional brain tissue, are necessary. The guidance of the biopsy needle by a navigation system warrants the necessary accuracy (1–7).

The depicted current method is relatively invasive, often time-consuming and carries several risks and side effects. In general, symptomatic intracerebral hemorrhages are reported in 1 – 10% of cases and are among the main risks because they may be associated with permanent morbidity or mortality (2, 3, 5, 8, 9). The reported rate of asymptomatic hemorrhages ranges from 8 – 54% (4, 8, 10). The accuracy of navigated brain biopsies is dependent on the used methodology and lies between 1 and 3 millimeters but may be impaired by the phenomenon of brain shift after the opening of the dura mater. The size of the bone opening, respectively, the opening of the dura mater and the duration of the procedure may be associated with the observed degree of brain shift (7, 11, 12). A further disadvantage of the current method is cosmetic impairment by a relatively large scar (13, 14).

The “proof of concept”-objective of this study is to answer the question if laser technology might be a suitable tool to make brain biopsies less invasive, perhaps also faster and safer. Laser technology might potentially enable us to miniaturize the necessary burr hole and also to angulate the burr hole much more tangential in relation to the bone surface in order to take biopsies from brain regions that are usually only difficult and hazardous to access.

We examined if it is possible to miniaturize the hole in the skull bone to such a high extent that potentially the laser-created canal itself may guide the biopsy needle with sufficient tolerance. The 2-dimensional, radial tolerance of the tip of the biopsy needles inserted in these canals was measured under defined lateral loads which mimic the mechanical forces applied by a surgeon during the procedure.

The use of pulsated Er : YAG lasers in bone ablation has been shown to produce precise hole geometries and chemical surface characteristics comparable to conventional drilling (15).

Due to the pulsation and the ER : YAG laser parameters in the “cold ablation” range (16), melting or carbonization typically associated with CO_2_-laser ablation can be avoided. Moreover, bone healing, under certain conditions, was found to be similar for osteotomies performed with Er : YAG lasers and piezoelectric osteotomes (17, 18). Bone healing might be improved in comparison to mechanical trepanation (10–21).

Furthermore, in contrast to conventional rotating drilling systems, lasers offer the possibility to create holes in pointed angles relative to the bone surface. The latter offers the opportunity to position the entry point of the biopsy needle in, e.g., hair-covered regions that are virtually invisible after the biopsy or to obtain biopsies from areas of the brain that are otherwise inaccessible or at least difficult and hazardous to reach.

Taken together, laser technology could have the potential to improve several aspects of the current practice of brain biopsies.

This proof of concept study examines if an Er : YAG laser can cut holes in different angles to the surface in native human skull bones with such a high level of precision that the created canal in the bone itself might act as a useable guide for the biopsy needle along the predefined trajectory. The radial tolerance of guidance of the tip of the inserted needle was measured under different predefined lateral loads applied to the “outside part” of the needle (i.e. at the outer surface of the skull bone) which mimic mechanical forces applied by a surgeon.

## Material and Methods

### Skull Bone

Vital human skull bone was immediately cryoconserved at -80° C after explantation. Frontal, temporal and parietal parts of human skull bones have been used to experiment with a realistic reconstruction of a usual brain biopsy setup. The thickness of the skull bone samples ranged from 1.5 - 11.2 millimeters. For the experiments, the skull bone samples were thawed to room temperature.

According to the responsible ethics committee, formal ethical approval was not required due to anonymized bone material from deceased donors.

### Laser Osteotome

To create the holes in the bone samples for the biopsy needle along predefined trajectories, the CARLO^®^-system (Cold Ablation Robot-guided Laser Osteotome; AOT, Basel, Switzerland) was used. CARLO^®^ consists of an Er : YAG laser which is mounted on a navigated multiaxial robotic arm. The used Er : YAG laser is designed to perform “cold ablation” with a pulse energy of 650 mJ and a pulse duration of 200 µs as described in (16, 22).

The Er : YAG laser-emitted light has a wavelength of 2943 nanometers with a pulse repetition frequency of 10 Hertz. The achievable bone ablation of the used laser osteotome is around 1 millimeter in depth and 0.8 millimeters in width per pulse.

### Experimental Setup

**Figure 1:**
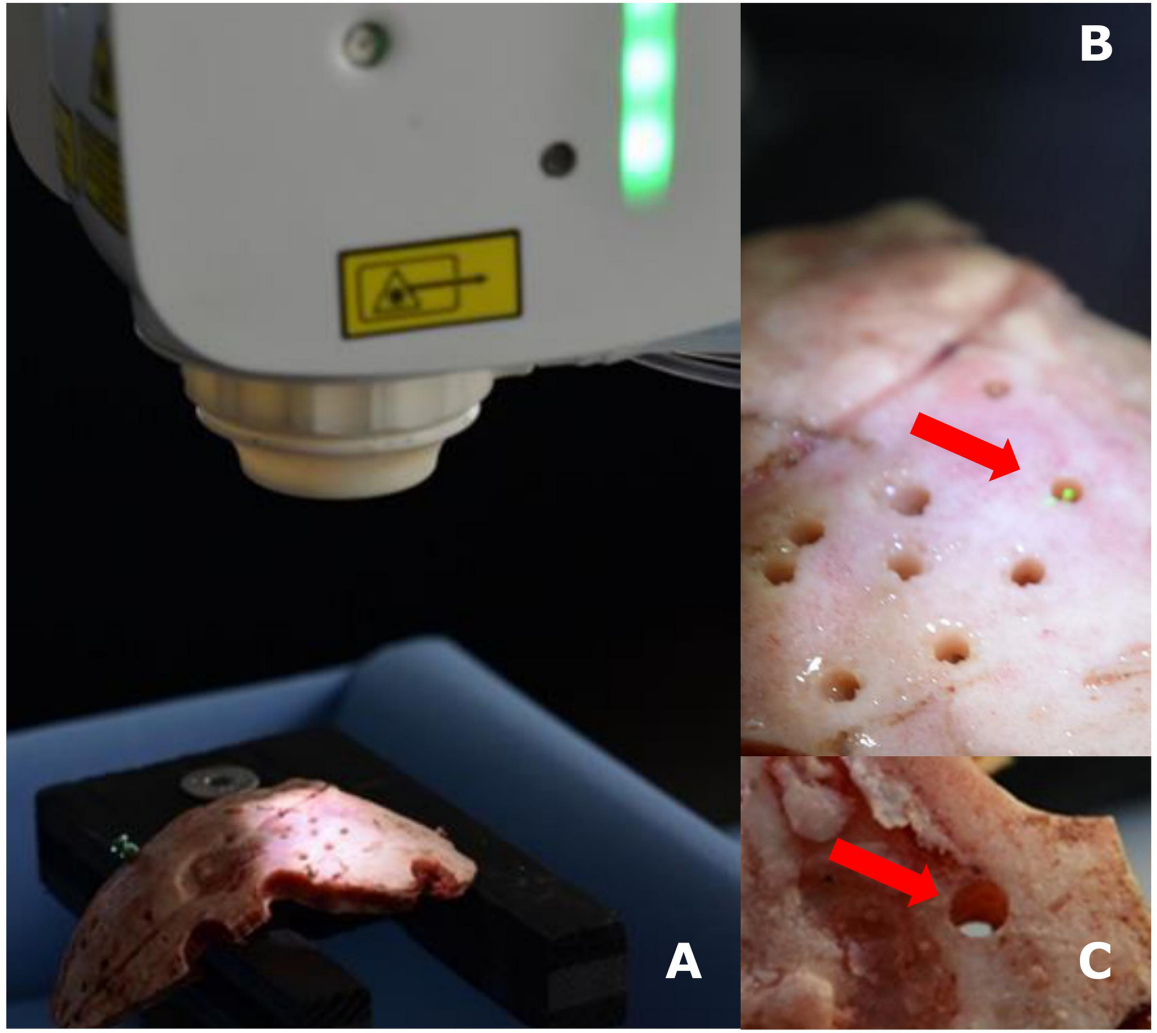
Experimental setup with navigated Er : YAG laser and skull bone **(A)**, detailed photos of the laser in action **(B)**, and a laser-created hole in skull bone **(C)**.

The skull bone samples were securely fastened on a workbench. The canals through the skull bones were planned in angles of 90° (perpendicular) and 45° relative to the bone surface. After referencing the navigation system, the robotic arm with the attached Er : YAG laser was programmed concerning the planned trajectories and the 1.8-millimeter diameter of the hole, i.e. the diameter of the biopsy needle (BrainPro, PAJUNK^®^, Germany), and activated (**Figures 1A–C**)

**Figure 2:**
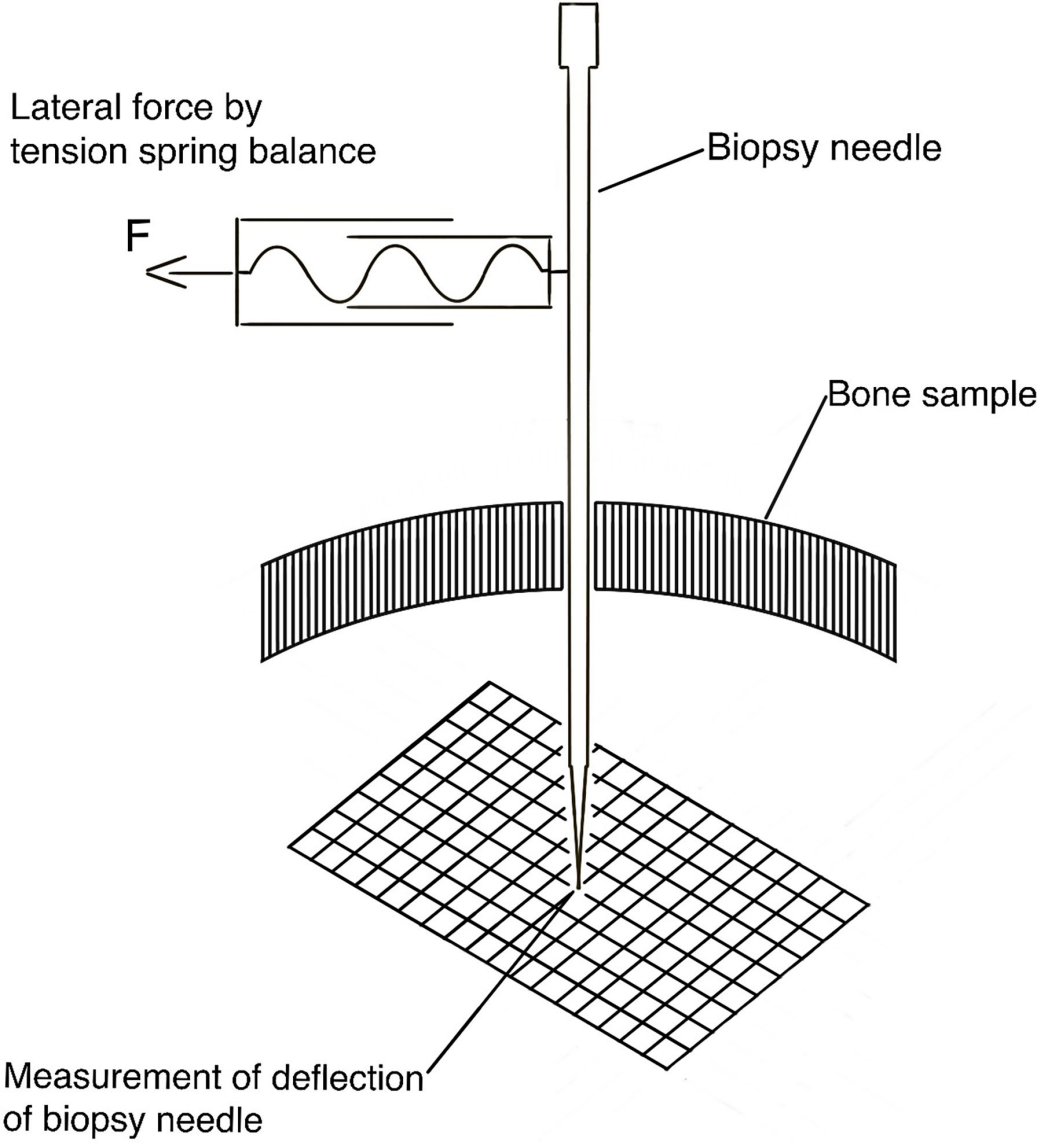
Schematic drawing of the experimental setup to measure the deflection of the tip of the biopsy needle under different lateral loads.

Following the creation of the hole, the biopsy needle was inserted up to a predefined depth (5.0 cm), and the axis of the biopsy needle was oriented perpendicularly to a scale paper with millimeter-division. By using a tension spring balance, lateral forces of 0.05 N, 0.1 N, 0.2 N, 0.5 N, and 1 N were applied to the proximal part (i.e. at the outer surface of the skull bone, 10.0 cm distance from the bone surface) of the biopsy needle and the correlating radial tolerance, as reflected by the measured deflection, of the tip of the biopsy needle was measured (**Figure 2**).

These applied lateral forces represent different forces during the handling of the biopsy needle by the neurosurgeon. The radial tolerance of the distal tip of the biopsy needle, i.e. at the inside of the skull bone at the different applied forces was then recorded by using the underlying scale paper with an accuracy of 0.5 millimeters.

## Results

In three skull bone samples, 33 holes were created by the Er : YAG laser. The planned diameter of the bone canals was 1.8 millimeters which equals the outer diameter of the biopsy needle. 13 holes had been created at an angle of 45°, 20 holes at an angle of 90° to the outer bone surface.

The first eight holes were utilized to identify the appropriate laser parameters to cut skull bone. These eight holes have not been used for the further examinations.

In all of the the next 25 holes, the biopsy needles could be introduced with low resistance with a perceptible defined guidance along the trajectory due to the holes’ exact fit. These 25 holes have been used for further examinations.

**Figure 3:**
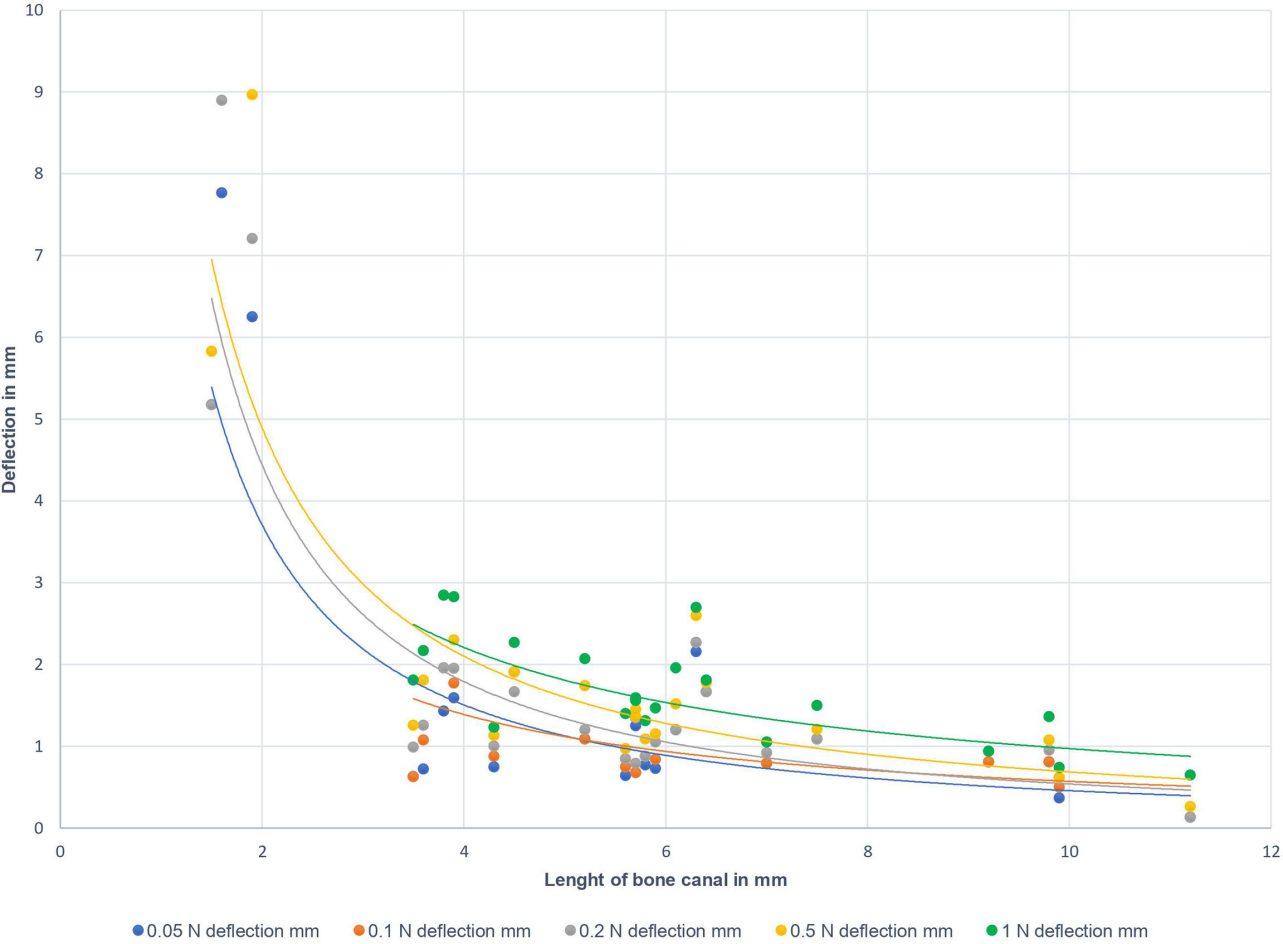
Deflection of the tip of the biopsy needle in 5.0 cm distance from the inner bone surface to the bone canal’s length. Different colors represent the applied lateral loads at the outer part of the biopsy needle (10.0 cm distance from outer bone surface): blue – 0.05 N; orange – 0.1 N; grey – 0.2 N; yellow – 0.5 N; green - 1.0 N.

In the following, measurement of the radial tolerance of the tip of the biopsy needle after lateral load application as described above, has been performed. An increase of the applied lateral force was generally associated with a slightly increased deflection of the tip of the biopsy needle. **Figure 3** displays the deflection of the tip of the biopsy needle at 5.0 cm distance from the skull bone sample’s inner surface. The colored dots represent the deflection at different defined lateral forces (0.05 N, 0.1 N, 0.2 N, 0.5 N, 1.0 N). Correlated to the applied loads, no differences in the deflection values were found for the holes in 45°- and 90°-orientation to the surface. A longer canal through the bone was associated with less deflection, i.e. a more accurate guidance of the needle along the trajectory. The bone canal’s length is influenced by the thickness of the bone and the angle of the hole in relation to the bone surface. It is notable that forces of more than 0.2 N cause a visible bending of the biopsy needle on the outside part of the needle, where the load was applied.

## Discussion

Brain biopsies are a routinely used diagnostic tool, but the usual current brain biopsy procedure is still a relatively invasive intervention that carries several disadvantages and risks.

The ongoing evolution of laser technology might carry the opportunity to improve the procedure of brain biopsies regarding aspects of invasiveness, safety and surgery time. Lasers have been shown to cut biologic tissue such as bone, fascia, or soft tissue with high precision and less thermal strain to adjacent tissue than mechanical bone drilling techniques and good or even superior postoperative wound healing (20, 21, 23, 24). Concerning Er : YAG lasers, the absorption of the transmitted energy is nearly exclusively by the water molecules in the tissue, resulting in only minimal thermal stress of surrounding tissue (21, 25, 26). In contrast to conventional drills, lasers do not leave metal debris at their site of action, which might produce artifacts in subsequent MR-imaging studies (22, 27).

Finally aiming at the further development of less invasive brain biopsy techniques, the presented study aims to evaluate as a first “proof of principle”-step if it is possible to miniaturize the hole in the skull bone to such a high extent that potentially the laser-created canal itself may guide the biopsy needle with acceptable tolerance.

A potential future scenario could be the use of high-precision holes in the skull bone as a guidance for the biopsy needle without the need for an additional and error prone mechanical navigated guiding system.

In our examinations, we at first established knowledge about high-precision cutting of the skull bone with an Er : YAG laser. Subsequently, with the adequate laser parameters, we found that the laser provided reproducible and exact fitting apertures in the skull bone to insert the biopsy needle. The needle was inserted to a depth of 5.0 cm distance to the inner surface of the skull bone. By applying defined lateral forces to the part of the biopsy needle at the skull’s outer surface, we simulated the manipulation of the needle by the neurosurgeon during a biopsy. A trained neurosurgeon will not exceed forces of 0.2 N during insertion resp. manipulation of the needle. However, we also examined excessive forces up to 1.0 N of lateral load. In 87 of 93 measurements, the deflection, i.e. the radial tolerance, of the tip of the biopsy needle was less than 3 millimeters.

Assuming a usual scenario with bone canal lengths of more than 4 millimeters and the expectable lateral loads during the procedure, the deflection of the tip of the biopsy needle was less than 2.5 millimeters. In clinical application, the accuracy of a mechanical navigated guiding system would be the equivalent to the observed radial tolerance.

Notably, the observed primary determinant for the biopsy needle’s deflection was the bone canal’s length and not the lateral force applied to the biopsy needle (see **Figure 3**). However, bone canal lengths of less than 3 millimeters did not provide sufficient guidance if one presumes that a maximum deviation of 3 millimeters in 5 centimeters distance from the inner skull surface is sufficient for the planned biopsy. This tolerance is within the range of reported accuracy-values of different conventional navigation systems (28).

The depicted results demonstrate the potential that lies in this technology. Regarding the whole procedure of brain biopsies, these results can be regarded as a first and groundbreaking step in the further development of brain biopsies that use laser technology. There are several other steps of the whole biopsy procedure that need to be re-thought. The necessary short skin incision itself could potentially also be created by a laser or, if using a conventional scalpel, at least substantially be shortened to introduce a small speculum-like device to keep the incision open. In practical use, when creating the canal through the skull bone by laser technology, the laser energy transmission needs to be terminated when the thickness of the skull bone is traversed, and the dura mater is the only remaining firm mechanical barrier to the brain. The technique of optical coherence tomography can terminate the transmission of laser energy at precisely this point and is part of current research (29, 30). A laser could also perform the puncture and hemostasis of the dura mater with adapted parameters. The corticotomy at the entry site of the biopsy needle in the brain could be done by laser or by electrocautery. After obtaining the biopsy, an appropriate laser could be employed to improve the subcutaneous wound closure. Regarding this wide range of potential applications, laser technology could be a tool to make the procedure of brain biopsies much faster because the usual multiple change of instruments becomes unnecessary. A shorter surgical procedure time and the smaller operative situs might lower the perioperative risk profile. Furthermore, the much smaller incision and smaller hole in the bone would improve patient satisfaction due to better cosmetic results.

## Conclusion

Laser technology might be a valuable tool to improve the procedure of brain biopsies regarding invasiveness, procedure time and finally risk profile. In this “proof of principle”-study with an Er : YAG laser we could show, as a prerequisite for further research, that this technology can create canals in skull bone with such a high degree of precision that these canals alone could act as a sufficient guide for the biopsy needles.

## Data Availability Statement

The raw data supporting the conclusions of this article will be made available by the authors, without undue reservation.

## Ethics Statement

The studies involving human participants were reviewed and approved by Ethics Committee of Northwest and Central Switzerland.

## Author Contributions

DC, initiation and realization of project, overall responsibility for project, literature work, planning of experimental setup, evaluation of results, manuscript compilation, and ethical issues. TH, planning of experimental setup, execution of experiments, evaluation of results, and literature work. MB, planning of experimental setup and execution of experiments. FT, project expertise, literature work, and revision of manuscript. All authors contributed to the article and approved the submitted version.

## Conflict of Interest

Author MB was employed by company Advanced Osteotomy Tools AG.

The remaining authors declare that the research was conducted in the absence of any commercial or financial relationships that could be construed as a potential conflict of interest.

## Publisher’s Note

All claims expressed in this article are solely those of the authors and do not necessarily represent those of their affiliated organizations, or those of the publisher, the editors and the reviewers. Any product that may be evaluated in this article, or claim that may be made by its manufacturer, is not guaranteed or endorsed by the publisher.
